# Burden of RSV in Young Children in High‐Income Countries: Incidence Estimates From a Multilevel Meta‐Analysis in Primary and Emergency Care

**DOI:** 10.1111/irv.70179

**Published:** 2025-10-26

**Authors:** Susanne Heemskerk, Lotte van Heuvel, Peter Spreeuwenberg, Louis J. Bont, Foekje F. Stelma, Saverio Caini, Jojanneke van Summeren

**Affiliations:** ^1^ Netherlands Institute for Health Services Research (Nivel) Utrecht the Netherlands; ^2^ Department of Paediatric Infectious Diseases and Immunology Wilhelmina Children's Hospital, University Medical Center Utrecht Utrecht the Netherlands

**Keywords:** child, emergency department, global burden of disease, primary health care, respiratory syncytial virus

## Abstract

**Background:**

Most respiratory syncytial virus (RSV) infections in children are managed in primary care settings, including ambulatory care and emergency departments (EDs). This study provides adjusted pooled RSV incidence estimates for children under 5 years in primary care settings in high‐income countries (HICs).

**Methods:**

We used population‐based RSV incidence rates from 27 studies collected in a previous systematic review as input parameters. To adjust for heterogeneity in study design, we assessed the impact of four key factors: 1) age, 2) primary care setting (ambulatory care or EDs), 3) data collection period (year‐round or seasonal), and 4) study methodology (cohort studies with laboratory‐confirmed RSV, healthcare databases, surveillance data). In the final model, we corrected for age, primary care setting, and study methodology. Adjusted pooled RSV incidence estimates were calculated using a multilevel logit‐logistic regression model.

**Results:**

For children < 5 years, the adjusted pooled RSV incidence estimate in primary care settings was 62.8 per 1000 population (95% CI 45.3–86.6). Incidence was higher in ambulatory care (108.1 per 1000; 95% CI 78.0–148.0) compared to EDs (35.8 per 1000; 95% CI 25.3–50.3). Age‐stratified incidence estimates declined with increasing age, showing 86.5 (95% CI 61.6–120.2), 80.3 (95% CI 57.1–111.8), 60.7 (95% CI 43.2–84.6), and 36.5 (95% CI 25.4–52.2) per 1000 for children aged < 6 months, 0–1 year, 0–2 years, and 0–5 years, respectively.

**Conclusions:**

This is the first multilevel meta‐analysis estimating the RSV‐related burden in primary care settings, including both ambulatory and emergency care. These results can be used by decision makers for the introduction of RSV immunization programs.

## 1 Introduction

Respiratory syncytial virus (RSV) is the leading cause of acute respiratory infections in young children [1]. The global burden of RSV in hospital settings has been extensively studied. In 2019, it was estimated that RSV caused 3.6 million hospitalizations, 26,300 in‐hospital deaths and 101,400 overall deaths among children under 5 years old worldwide [2]. In Europe, approximately 245,244 yearly hospital admissions associated with RSV in children under 5 years were estimated, with the majority of cases occurring in infants under 1 year of age [3]. Additionally, approximately 1 in 7 healthy term‐born infants in Europe seek medical care for RSV in their first year of life, with 1 in 56 requiring hospitalization [4].

Since 2022, European and American regulatory agencies have approved two novel preventive measures for infants against RSV [5]. These include nirsevimab, a single‐dose long‐acting monoclonal antibody, and the RSVpreF vaccine, a single‐dose vaccine administered to pregnant women to protect newborns [6, 7]. Additionally, other preventive measures targeting toddlers are currently in clinical development [5]. To support policymakers in decision‐making regarding the introduction of these new measures in national vaccination programs, more detailed knowledge is needed on the burden of RSV in different healthcare settings.

Although severe RSV infections often require hospitalization [1, 2, 3, 4], most RSV cases are managed in primary care settings [8]. These primary care settings—including ambulatory care settings such as general practitioners, pediatric offices and outpatient clinics, as well as emergency departments (EDs)—typically serve as the initial point of contact for most children seeking medical care for RSV. A study conducted in five European countries found that RSV infections in young children managed in primary care are linked to significant symptom burden, healthcare utilization, and parental work absence [9]. However, the global burden of RSV in primary care settings remains poorly understood. Incidence estimates for RSV‐associated primary care visits remain limited, largely due to the absence of routine RSV testing in these primary care settings [10]. To address this gap, some studies use clinical diagnoses of bronchiolitis as a proxy for RSV, given that RSV is a common cause of bronchiolitis, while others rely on routinely collected surveillance data to estimate RSV‐associated primary care visits [11, 12, 13].

To improve understanding of the RSV burden in primary care settings, a recent systematic literature review synthesized population‐based incidence rates of RSV infections and bronchiolitis in children under five in ambulatory and emergency care settings [14]. However, substantial heterogeneity was observed across studies due to differences in study design factors such as age groups, care settings, data collection periods, and study methodologies. Consequently, producing reliable pooled incidence estimates without accounting for these variations was not possible. The aim of this study is to adjust for the observed heterogeneity in study design factors and generate robust pooled incidence estimates of RSV infections in primary care settings—including ambulatory care and EDs—among children under 5 years in high‐income countries (HICs).

## 2 Methods

### 2.1 Input Data

This study used yearly RSV incidence rates reported in studies collected in our previously published systematic literature review as input data [14]. Full details of the search strategy, inclusion criteria and study selection process are available in the published review. A total of 27 studies from HICs were included in this review, providing incidence rates for 10 countries. For studies reporting incidence rates across multiple seasons, the mean incidence rate of these seasons was used. When studies reported incidence rates for different age groups or primary care settings, multiple estimates from the same study were included. In total, 58 incidence estimates from 27 studies were used as input for the meta‐analysis. The reported RSV incidence rates in the included studies ranged from 0.8 to 330 per 1000 population in ambulatory care and from 7.5 to 144 per 1000 in EDs.

To adjust for heterogeneity in study design factors, the impact of four key factors was assessed: 1) age group, 2) primary care setting (ambulatory care or ED), 3) data collection period (year‐round or seasonal), and 4) study methodology. Study methodology was categorized into three groups: a) cohort and registry database studies only including laboratory‐confirmed RSV cases, b) surveillance‐based studies and modeling studies using surveillance data, and c) electronic health record database studies (RSV not necessarily laboratory‐confirmed). Age groups were categorized into the most appropriate age groups based on the age ranges reported in the includes studies, using the following nested categories: <6 months, 0–1 year, 0–2 years, and 0–5 years. Consequently, younger children are included in multiple categories, which may lead to their overrepresentation in our overall study population.

### 2.2 Statistical Analyses

We used a multilevel (meta‐analysis) logit‐logistic regression model. This method was previously described by Paget et al. 2023 [15]. The primary outcome measure (dependent variable) was the yearly RSV incidence estimate per 1000 population in primary care settings in children aged < 5 years. The fixed estimate of the model was the overall average RSV incidence rate; similar to the overall rate in other forms of meta‐analysis. The model consisted of three levels (random effects), accounting for variability and differences between outcomes. Level 1 represented the individual estimates per study, where binomial error variance (constrained to 1) was modeled. Level 2 modeled the variance between individual measurements within a study and level 3 modeled between‐study variance. See Supplementary Material I for the statistical multilevel meta‐analysis model. The four study design factors—age group, primary care setting, data collection period, and study methodology—were added to the model to determine the impact on reported incidence estimates. Every category in a study design factor was given an equal weight (indicator scoring (0,1) minus 1/*N* (cat), *N* (cat) = number of categories in factor). This means that all age groups were weighted equally, with the < 6 months age group receiving the same weight as the 0–5 years age group. The final model was adjusted for study design factors substantially impacting the incidence estimates. Subgroup analyses were performed to generate pooled incidence estimates for ambulatory care and EDs, and children aged < 6 months, 0–1 year, 0–2 years, and 0–5 years.

All models were estimated in MLwiN version 3.01 using restricted iterative generalized least squares (RIGLS) with first‐order penalized quasi‐likelihood (PQL). No weight was assigned based on sample size due to the significant heterogeneity between individual measures and studies.

## 3 Results

### 3.1 Characteristics of the Included Studies

Study characteristics and RSV incidence rates are described in Table 1. All studies were conducted before the COVID‐19 pandemic, with data collected between 1992 and 2019. The studies were conducted in Europe (*n* = 10), North America (*n* = 15), and Oceania (*n* = 2). In 26 studies, incidence estimates were calculated for a single country, while one European study reported incidence rates for two countries (England and the Netherlands). Eleven studies reported incidence rates for multiple age groups under 5 years. Fifteen studies reported incidence rates in ambulatory care and 16 in EDs, with four studies providing incidence rates for both. Stratified by study methodology, there were eight cohort studies only including laboratory confirmed RSV cases, nine surveillance‐based studies, and 10 healthcare database studies.

**TABLE 1:** Input data for multilevel analyses (ordered by country a‐z).

Author	Year data	Country	Study methodology^a^	Data collection period	Care setting	Age	Yearly RSV incidence rate per 1000 population (95% CI^b^)
**Europe**							
Cromer et al. (2017) [16]	2017	England	Surveillance (m)	Year‐round	Ambulatory care	0–5 months	214.2 (211.3–217)
						0–59 months	119.9 (118.6–121.2)
Paget (2010)a [17]	2002–2008	England	Surveillance (m)	Seasonal	Ambulatory care	0–59 months	0.8
Heikkinen (2017) [18]	2000–2002	Finland	Cohort	Seasonal	Ambulatory care	0–11 months	263 (133–479)
						0–23 months	263 (153–431)
						0–59 months	212 (133–327)
Thomas (2021) [19]	2017–2018	Finland	Cohort	Seasonal	Ambulatory care	0–3 months	328.4 (275.2–389.0)
Forster (2004) [20]	1999–2001	Germany	Cohort	Year‐round	Ambulatory care	0–35 months	77 (67–89)
Barbieri (2023) [21]	2012–2019	Italy	Healthcare database	Year‐round	Ambulatory care	0–5 months	111.9 (108.3–115.6)
						0–11 months	89.9 (87.7–92.0)
						0–24 months	46.6 (45.5–47.6)
				Seasonal	Ambulatory care	0–24 months	76.5 (74.7–78.4)
Dolk (2021) [22]	2003–2014	Netherlands	Surveillance	Seasonal	Ambulatory care	0–59 months	17.6
Paget (2010)b [17]	2002–2008	Netherlands	Surveillance (m)	Seasonal	Ambulatory care	0–59 months	3.8
Bueno Campaña (2008) [23]	2003	Spain	Cohort	Seasonal	Ambulatory care	0–5 months	119
Muñoz‐Quiles (2016) [24]	2009–2012	Spain	Healthcare database	Year‐round	Ambulatory care	0–5 months	190
						0–11 months	197
						0–23 months	143
Reyes Dominguez (2022) [25]	2016–2019	Spain	Cohort^a^	Year‐round	ED	0–24 months	53.4
**North America**							
Brunet (2022) [26]	2004–2018	Canada	Database	Year‐round	ED	0–23 months	29.1 (28.9–29.3)
Law (2004) [27]	2001–2003	Canada	Cohort	Seasonal	ED	0–6 months	82.7
Rosychuk (2011) [28]	1999–2005	Canada	Healthcare database	Year‐round	ED	0–35 months	38.2
To (2022) [29]	2008–2019	Canada	Healthcare database	Year‐round	ED	0–11 months	20.7
						0–59 months	7.5
Ambrose (2014) [30]	2009–2011	USA	Cohort	Seasonal	Ambulatory care	0–6 months	330 (279–388)
					ED	0–6 months	141 (108–179)
Bourgeois (2006) [31]	1993–2004	USA	Surveillance	Seasonal	ED	0–5 months	144.0 (140–148)
						0–23 months	75.7 (73.8–77.7)
						0–59 months	40.4 (39.3–41.5)
Bourgeois (2009) [32]	2003–2005	USA	Cohort	Seasonal	ED	0–23 months	64.4 (45.4–91.3)
Hall (2009) [33]	2002–2004	USA	Surveillance	Seasonal	Ambulatory care	0–5 months	132 (46–383)
						0–11 months	155 (54–447)
						0–23 months	110 (36–346)
						0–59 months	80 (36–179)
					ED	0–5 months	55 (24–126)
						0–11 months	56 (22–144)
						0–23 months	44 (17–118)
						0–59 months	28 (15–50)
Hasegawa (2014) [34]	2006 & 2010	USA	Healthcare database	Year‐round	ED	0–11 months	53.9
						0–23 months	35.9
Jackson (2021) [35]	2018–2019	USA	Surveillance	Seasonal	Ambulatory care	12–23 months	107
						24–59 months	60
Lively (2019) [36]	2004–2009	USA	Surveillance	Seasonal	Ambulatory care	0–5 months	215.7 (179.8–251.5)
						0–11 months	230.9 (192.5–269.3)
						0–23 months	205.7 (169.5–241.9)
					ED	0–5 months	74.8 (64.0–85.6)
						0–11 months	66.2 (56.6–75.7)
						0–23 months	59.6 (50.9–68.3)
Mansbach (2005) [37]	1992–2000	USA	Healthcare database	Year‐round	ED	0–23 months	26 (22–31)
Mansbach (2007) [38]	1993–2004	USA	Healthcare database	Year‐round	Ambulatory care	0–23 months	103 (83–124)
Rainisch (2020) [39]	2002–2009	USA	Surveillance (m)	Year‐round	Ambulatory care	0–11 months	230.9 (71.0–337.2)
					ED	0–11 months	66.2 (16.8–132.7)
Suh (2022) [40]	2011–2019	USA	Healthcare database	Year‐round	ED	0–11 months	33.8 (31.7–35.9)
**Oceania**							
Moore (2012) [41]	2001–2005	Australia	Healthcare database	Year‐round	ED	0–5 months	59.7
						0–11 months	51.0
						0–23 months	29.6
						0–59 months	11.8
Prasad (2020) [42]	2014–2016	New Zealand	Surveillance	Seasonal	ED	0–11 months	34.4 (32.1–36.7)

Abbreviations: CI, confidence interval; ED, emergency department; RSV, respiratory syncytial virus.

^a^ “Cohort” in this study includes cohort studies based on laboratory‐confirmed RSV cases and one healthcare database study only including laboratory‐confirmed RSV cases (indicated with ^a^); “Surveillance” includes surveillance‐based studies and modeling studies using surveillance data, the modeling studies are indicated with (m); “Healthcare database” includes studies based on electronic health records such as ICD‐9 or ICD‐10 codes (RSV not laboratory‐confirmed).

^b^ Only included for studies that report the 95% CI.

### 3.2 RSV Incidence Estimates

The multilevel model outcomes are presented in Table 2. The fixed effects analysis revealed that RSV incidence rates were significantly impacted by age and care setting, with study methodology also showing a considerable effect (although not significant). Therefore, in the final model, RSV incidence estimates were adjusted for age, primary care setting, and study methodology.

**TABLE 2:** Multilevel model outcomes for children < 5 years.

Fixed effect			
Independent factors	Estimate	SE	*p*
Intercept	−2.696	0.180	<0.001
Age *(< 6 months compared to 0–5 years)*	0.919	0.110	<0.001
Age *(0–1 year compared to 0–5 years)*	0.838	0.112	<0.001
Age *(0–2 years compared to 0–5 years)*	0.531	0.107	<0.001
Setting *(ambulatory care compared to EDs)*	1.185	0.091	<0.001
Data collection period *(seasonal compared to year‐round data)*	0.069	0.217	0.752
Method *(cohort compared to healthcare database)*	0.668	0.456	0.143
Method *(surveillance compared to healthcare database)*	−0.350	0.440	0.427

Random effects			
Level	Estimate	SE	*p*
Measurement outcome (level 2)	0.027	0.010	0.005
Study (level 3)	0.768	0.216	<0.001

Abbreviations: EDs, emergency departments; SE, standard error.

Table 3 presents the RSV incidence estimates for children under 5 years in HICs. The overall incidence estimate in primary care settings is 62.8 per 1000 population (95% CI 45.3–86.6), and is significantly higher in ambulatory care (108.1 per 1000; 95% CI 78.0–148.0) compared to EDs (35.8 per 1000; 95% CI 25.3–50.3). Age‐stratified estimates show the highest RSV incidence in infants aged < 6 months (86.5 per 1000; 95% CI 61.6–120.2), with a gradual decline to 80.3 (95% CI 57.1–111.8) in the 0–1 year age group, 60.7 (95% CI 43.2–84.6) in the 0–2 year group, and 36.5 per 1000 (CI% 25.4–52.2) in children aged 0–5 years.

**TABLE 3:** Adjusted pooled RSV incidence estimates in primary care settings.

	Incidence estimate per 1000	95% CI
Primary care settings	62.8	45.3–86.6
*Stratified by care setting*		
Ambulatory care	108.1	78.0–148.0
EDs	35.8	25.3–50.3
*Stratified by age*		
<6 months	86.5	61.6–120.2
0–1 year	80.3	57.1–111.8
0–2 years	60.7	43.2–84.6
0–5 years	36.5	25.4–52.2

Abbreviations: CI, confidence interval; EDs, emergency departments; RSV, respiratory syncytial virus.

## 4 Discussion

This is the first study to provide pooled incidence estimates for RSV infections in primary care settings among children under 5 years in HICs. The overall estimated RSV incidence was 62.8 per 1000, with rates around three times higher in ambulatory care compared to EDs. RSV primarily affects infants and young children in primary care settings, with the highest incidence observed in those under 1 year of age, although it remains notably prevalent among all children under 5 years.

Understanding RSV burden in children under 5 years in primary care settings is crucial to support immunization policies. Compared to other healthcare settings, the estimated incidence in primary care is higher than community‐based estimates (24.3 per 1000) and substantially exceeds hospital‐based estimates in HICs (6.0 per 1000) [2]. Age‐specific comparisons show the same pattern: incidence in infants < 6 months was 86.5 per 1000 in primary care and 28.4 per 1000 in hospitals, while in children aged 0–1 years it was 80.3 and 22.0 per 1000, respectively [2]. Moreover, a recent study showed that RSV infections in primary care contribute to considerable healthcare and societal costs in Europe [43]. These findings underline the broad public health and economic impact of RSV in primary care settings.

Because healthcare systems are organized differently across the world, immunization strategies must be tailored to each country's specific needs. For example, pediatric primary care is provided by general practitioners or pediatricians in Europe, while in the USA, children with RSV are commonly seen in outpatient clinics or EDs during initial medical contact [44, 45]. This variation in healthcare organization was also reflected in our study, resulting in only one European study being conducted in an ED setting [25]. Importantly, because most ED data came from North America and most ambulatory data from Europe, the higher incidence estimates in ambulatory care compared to EDs may not only reflect setting‐specific burden but also regional differences in healthcare organization and testing practices. These differences highlight the importance of using country‐specific RSV data to inform RSV immunization policy. Alternatively, estimates from countries with similar healthcare structures may serve as proxies where national data are lacking.

Future research should focus on expanding the geographical scope of RSV incidence studies in primary care to generate more robust and globally representative estimates. In our meta‐analysis, data from the WHO Western Pacific region and from Northern and Eastern Europe were scarce. As a result, the generalizability of our pooled estimates is largely limited to specific high‐income regions, particularly Western Europe and North America. Equally important are the limited number of studies from low‐ and middle‐income countries (LMICs), despite evidence that these regions carry the highest RSV burden and account for the majority of RSV‐related deaths [1, 2]. In our previous literature review, only 5 out of the 32 studies were conducted in LMICs [14], and these few studies reported lower primary care incidence rates compared with HICs. Similar patterns have been observed in hospital incidence, with lower rates in low‐income countries (3.5 per 1000) compared with HICs (6.0 per 1000), while middle‐income countries reported incidence rates comparable to HICs (6.2 per 1000 vs. 6.0 per 1000) [2]. This apparent discrepancy likely reflects limited healthcare access and differences in healthcare‐seeking behavior. Increasing research in underrepresented regions is therefore essential to improve understanding of the global burden of RSV in primary care and to generate more accurate global incidence estimates.

Our methodological approach was adapted from a previous multilevel meta‐analysis on influenza‐associated hospitalizations [15], which identified key factors of heterogeneity such as laboratory confirmation and the number of seasons included. While our study followed a similar approach, it was based on a smaller dataset (27 studies vs. 127 in the influenza study), limiting our ability to explore additional influencing factors. Expanding the number of included studies in future analyses will allow for a more comprehensive evaluation of heterogeneity in primary care settings, ultimately leading to more robust and better‐adjusted pooled estimates. A larger dataset would also enable the generation of regional incidence estimates, which is relevant given previous findings indicating variations in RSV disease burden across primary care settings in different European countries [9]. Additionally, incidence estimates for more granular age groups (e.g., <6 months, 6–12 months, 12–24 months) would be valuable, particularly in light of new RSV immunizations targeting specific pediatric age groups. Finally, as all included studies were conducted before the COVID‐19 pandemic, our estimates do not capture subsequent disruptions in RSV circulation [46, 47], changes in healthcare‐seeking behavior, modifications in surveillance practices, or the recent introduction of new immunization strategies. Repeating our methodology with postpandemic data would therefore provide valuable insights into the current incidence of RSV in primary care under these altered circumstances.

A few limitations should also be considered when interpreting the findings of this study. The incidence estimates are confined to the data available from the included studies and do not capture the heterogeneity in care settings worldwide. Consequently, the primary limitation of this analysis is the limited generalizability of the estimated RSV incidence. Another related limitation is the substantial heterogeneity in data collection methods across included studies. Furthermore, the use of nested age groups in the analyses—where, for example, children under 6 months are also part of the broader 0‐ to 5‐year category—may result in a disproportionate influence of younger age groups on the overall incidence estimate, potentially skewing it upwards. Lastly, the collinearity between care setting and geographical region limited our ability to determine whether observed differences reflected true setting‐specific effects or underlying regional variation.

In conclusion, RSV incidence estimates in primary care settings in HICs are substantial across the entire under 5‐year age group, with higher rates observed in ambulatory care compared to EDs. The burden is highest among infants and declines gradually with age. Expanding the number of studies on RSV incidence in primary care, covering a broader geographical scope and using standardized study design, would enhance data availability for future meta‐analyses, allowing for the calculation of global and regional burden estimates.

## Author Contributions

**Susanne Heemskerk:** conceptualization, methodology, data curation, writing – original draft, writing – review and editing, visualization, formal analysis. **Lotte van Heuvel:** conceptualization, data curation, writing – review and editing, methodology. **Peter Spreeuwenberg:** conceptualization, methodology, formal analysis, writing – review and editing, data curation. **Louis J. Bont:** writing – review and editing. **Foekje F. Stelma:** writing – review and editing, supervision. **Saverio Caini:** methodology, conceptualization, writing – review and editing, supervision. **Jojanneke van Summeren:** conceptualization, methodology, data curation, supervision, writing – review and editing, visualization.

## Conflicts of Interest

SH, LvH, PS, SC, FFS, and JvS declare that Nivel has received unrestricted grants from WHO, Sanofi, GSK, and the Foundation for Influenza Epidemiology outside the submitted work and that Nivel has received a grant from the Preparing for RSV Immunisation and Surveillance in Europe (PROMISE) project of the “Innovative Medicines Initiative 2 Joint Undertaking” Grant Agreement No. 101034339. This Joint Undertaking gets support from the “European Union's Horizon 2020 research and innovation programme” and the “European Federation of Pharmaceutical Industries and Associations”. LJB has regular interaction with pharmaceutical and other industrial partners. He has not received personal fees or other personal benefits. University Medical Centre Utrecht (UMCU) has received major funding (> €100,000 per industrial partner) for investigator‐initiated studies from AbbVie, MedImmune, AstraZeneca, Sanofi, Janssen, Pfizer, MSD, and MeMed Diagnostics. UMCU has received major funding for the RSV GOLD study from the Bill & Melinda Gates Foundation. UMCU has received major funding as part of the public‐private partnership IMI‐funded RESCEU and PROMISE projects with partners GSK, Novavax, Janssen, AstraZeneca, Pfizer, and Sanofi. UMCU has received major funding by Julius Clinical for participating in clinical studies sponsored by MedImmune and Pfizer. UMCU received minor funding (€1000–25,000 per industrial partner) for consultation and invited lectures by AbbVie, MedImmune, Ablynx, Bavaria Nordic, MabXience, GSK, Novavax, Pfizer, Moderna, AstraZeneca, MSD, Sanofi, Genzyme, and Janssen. LJB is the founding chairman of the ReSViNET Foundation.

## Peer Review

The peer review history for this article is available at https://www.webofscience.com/api/gateway/wos/peer‐review/10.1111/irv.70179.

## Supporting information


**Data S1:** Supporting information.

## Data Availability

All data used in this meta‐analysis are available within this article and in our previously published systematic review on RSV burden in primary and emergency departments [14].
