# Supplementary material for: Burden of RSV in Young Children in High‐Income Countries: Incidence Estimates From a Multilevel Meta‐Analysis in Primary and Emergency Care
Source: Influenza Other Respir Viruses. 2025 Oct 26;19(10):e70179. doi: 10.1111/irv.70179 (PMC12554624; doi:10.1111/irv.70179)
Supplement: Supplementary file 1 — Data S1: Supporting information. [file IRV-19-e70179-s001.docx]

**Supplementary materials**

***I. Statistical multilevel meta-analysis model***

The meta-analysis employs a multilevel framework with binary outcomes. This approach offers flexibility to account for the heterogeneity in study designs of studies and to distinguish different factors that influence the outcome. For a more comprehensive discussion of these models, please refer to the literature cited below.

$$Y_{i(j,k)}=\beta_{0}+\sum_{r=1}^{4} \beta_{r}X+\mu_{i}+\mu_{j}+\epsilon_{i(j,k)}$$

I = individual measurement

J = study level

K = country (cross-classified) level

Y_𝑖(𝑗,𝑘)_ = outcome measure, incidence rate per 1000 population

𝛽_0_ = intercept, modeled average incidence rate

𝛽_𝑟_X = fixed effects for the four factors (r), coding, (0/1) –(1/N_(categories)_)

𝜇_𝑖_ = between measurement variance

𝜇_𝑗_ = between study variance

𝜖_𝑖(𝑗,𝑘)_ = binomial error variance, constrained to 1

Note: In this model, studies are not weighted as is common in many meta-analyses. This decision is due to the considerable variability in study design and quality within the literature, making it impossible to assign meaningful weights.

Literature:

Fernández-Castilla B, Maes M, Declercq L, Jamshidi L, Beretvas S.N, Onghena P, Van den Noortgate W. A demonstration and evaluation of the use of cross-classified random-effects models for meta-analysis. *Behavior Research Methods* (2019) 51:1286-1304.

Paget J, Staadegaard L, Wang X, Li Y, van Pomeren T, van Summeren J, Dückers M, Chaves SS, Johnson EK, Mahé C, Nair H, Viboud C, Spreeuwenberg P. Global and national influenza-associated hospitalisation rates: Estimates for 40 countries and administrative regions. *Journal of global health* (2023) 13:04003.

Turner R.M, Omar R.Z, Yang M, Goldstein H, Thompson S.G. A multilevel model framework for meta-analysis of clinical trials with binary outcomes. *Statistics in Medicine* (2000) 19:3417-3432
